# Variations in Multiple Birth Rates and Impact on Perinatal Outcomes in Europe

**DOI:** 10.1371/journal.pone.0149252

**Published:** 2016-03-01

**Authors:** Anna Heino, Mika Gissler, Ashna D. Hindori-Mohangoo, Béatrice Blondel, Kari Klungsøyr, Ivan Verdenik, Ewa Mierzejewska, Petr Velebil, Helga Sól Ólafsdóttir, Alison Macfarlane, Jennifer Zeitlin, Euro-Peristat Scientific Committee

**Affiliations:** 1 THL National Institute for Health and Welfare, Helsinki, Finland; 2 TNO, Netherlands Organisation for Applied Scientific Research, Department Child Health, Leiden, The Netherlands; 3 Anton de Kom University of Suriname, Faculty of Medical Sciences, Department Public Health, Paramaribo, Suriname; 4 INSERM, Obstetrical, Perinatal and Paediatric Epidemiology Research Team, Centre for Epidemiology and Biostatistics (U1153), Paris-Descartes University, Paris, France; 5 Department of Global Public Health and Primary Care, University of Bergen and Medical Birth Registry of Norway, Norwegian Institute of Public Health, 5018, Bergen, Norway; 6 Ljubljana University Medical Center, Ljubljana, Slovenia; 7 Department of Epidemiology, National Research Institute of Mother and Child, Warsaw, Poland; 8 Institute for the Care of Mother and Child, Prague, Czech Republic; 9 Department of Obstetrics and Gynaecology, Landspitali University Hospital, Reykjavik, Iceland; 10 Centre for Maternal and Child Health Research, School of Health Sciences, City University London, London, Great Britain; Hôpital Robert Debré, FRANCE

## Abstract

**Objective:**

Infants from multiple pregnancies have higher rates of preterm birth, stillbirth and neonatal death and differences in multiple birth rates (MBR) exist between countries. We aimed to describe differences in MBR in Europe and to investigate the impact of these differences on adverse perinatal outcomes at a population level.

**Methods:**

We used national aggregate birth data on multiple pregnancies, maternal age, gestational age (GA), stillbirth and neonatal death collected in the Euro-Peristat project (29 countries in 2010, N = 5 074 643 births). We also used European Society of Human Reproduction and Embryology (ESHRE) data on assisted conception and single embryo transfer (SET). The impact of MBR on outcomes was studied using meta-analysis techniques with random-effects models to derive pooled risk ratios (pRR) overall and for four groups of country defined by their MBR. We computed population attributable risks (PAR) for these groups.

**Results:**

In 2010, the average MBR was 16.8 per 1000 women giving birth, ranging from 9.1 (Romania) to 26.5 (Cyprus). Compared to singletons, multiples had a nine-fold increased risk (pRR 9.4, 95% Cl 9.1–9.8) of preterm birth (<37 weeks GA), an almost 12-fold increased risk (pRR 11.7, 95% CI 11.0–12.4) of very preterm birth (<32 weeks GA). Pooled RR were 2.4 (95% Cl 1.5–3.6) for fetal mortality at or after 28 weeks GA and 7.0 (95% Cl 6.1–8.0) for neonatal mortality. PAR of neonatal death and very preterm birth were higher in countries with high MBR compared to low MBR (17.1% (95% CI 13.8–20.2) versus 9.8% (95% Cl 9.6–11.0) for neonatal death and 29.6% (96% CI 28.5–30.6) versus 17.5% (95% CI 15.7–18.3) for very preterm births, respectively).

**Conclusions:**

Wide variations in MBR and their impact on population outcomes imply that efforts by countries to reduce MBR could improve perinatal outcomes, enabling better long-term child health.

## Introduction

The number of multiple pregnancies is rising as a proportion of all pregnancies. The rising age at childbirth is increasing the rate of spontaneous multiple pregnancy as well as the use of assisted conception [1–3]. Multiple pregnancies carry higher risks of adverse fetal and neonatal outcomes and this has consequences for child health as well as for families and the health care system. Compared with singletons, babies from multiple pregnancies have substantially higher rates of preterm birth, perinatal mortality and longer term neuro-developmental impairments [4–6]. Higher mortality among multiples is largely due to preterm birth, but multiples also have higher rates of stillbirth at all gestational ages and higher neonatal mortality at term [7].

Preterm babies (born at <37 completed weeks of pregnancy) have higher mortality, morbidity and risk of impaired motor and cognitive development in childhood than babies born at term. Babies born before 32 completed weeks of gestation face the highest risks of adverse outcomes [8]. Studies comparing the long-term health of very preterm multiples and singletons are scarce, but show that twins are as likely, and may be even more likely, to experience long-term neurodevelopmental impairment than singletons. In the French EPIPAGE cohort, very preterm twins had lower Mental Processing Composite (MPC) scores than singletons and some twin-specific complications, such as discordant birth weight, were correlated with poor outcome [9]. Moderate preterm birth (32 to 36 weeks of gestation) is also associated with poor outcomes at birth [10, 11] and in childhood [12]. Preterm birth predisposes to higher risks of chronic diseases and mortality later in life [13, 14], but differences between singletons and twins have not been explored.

Socio-demographic and policy factors differ between European countries and can impact multiple pregnancy rates. Principally, European countries have different cultures, legislation and methods of funding assisted conception. The average number of embryos transferred and extent to which single embryo transfer (SET) is used varies by country [15]. European countries also vary widely in age at childbearing and this has implications for multiple birth rates [16].

In this study, we aimed to describe differences in multiple birth rates in European countries, to examine trends and clusters among them and to investigate the extent to which these differences contribute to adverse perinatal outcomes at a country level.

## Methods

### Data

Aggregated birth data were collected for the years 2004 and 2010 as part of the Euro-Peristat project that was set up to develop a set of indicators for monitoring and reporting perinatal health in Europe. In 2004, 26 countries (25 EU member states and Norway) participated in the project and in 2010, this increased to 29 (all 27 EU member states at the time except Bulgaria, and in addition Iceland, Norway and Switzerland)[17–19]. In most countries the data come from medical birth registers, civil registration and child health systems. Data provision is largely mandatory and the coverage is good, but these registers are voluntary in Malta and Belgium. France, Cyprus, and Spain conduct surveys to monitor births and perinatal care to complement routinely collected administrative data. Data collection methods were described in fuller detail in previous articles [19, 20] and in the European Perinatal Health Reports[17, 21].

The data collected included tabulations of the number of deliveries by number of fetuses (singleton, twin, triplet, quadruplet or more) as well as the numbers of live births, fetal deaths and neonatal deaths by gestational age in completed weeks separately for singletons and multiples. Euro-Peristat requests gestational age data using the best obstetrical estimate but not all countries were able to state how gestational age is estimated. We also used data collected for the Euro-Peristat indicator of maternal age to describe the proportion of mothers aged 35 years or more.

All but two countries (Greece and Hungary) participating in the 2010 data collection were able to submit data about the number of multiple births and gestational age. Data about neonatal deaths by multiplicity were not available in Germany, Greece, Spain, Hungary, France and Cyprus. In Belgium, Brussels, Flanders and Wallonia contributed data separately as did England and Wales, Northern Ireland and Scotland in the UK. In Belgium and the UK, we combined data from different sources to describe multiple birth rates. However, as outcome data came from different sources, these data are also presented separately. Data on maternal age were available for all countries. Data for Cyprus refer to 2007.

In addition to these indicators we used data for 2010 from the European Society of Human Reproduction and Embryology (ESHRE) about the proportions of babies born following assisted conception and SET [22]. ESHRE data are aggregated data on procedures in individual centers in each country, and only countries with 100% coverage are shown. However, the country of residence of the parents and where they deliver is unknown. The number of live births after assisted conception collected by ESHRE is based on year of procedure while Euro-Peristat data is based on the year of birth.

This study was based on aggregated routinely collected data, so ethics approval was not required.

### Definitions of outcome variables

We calculated multiple birth rates for countries in 2004 and 2010 as the number of women with multiple pregnancies (twin and triplet or more) per 1000 women delivering a live or stillbirth.

The analysis of health outcomes was carried out on data from 2010 separately for singletons and multiples using four perinatal health indicators: preterm birth (number of live births with a gestational age <37 weeks per 100 live births), very preterm birth (number of live births with a gestational age <32 weeks per 100 live births), fetal mortality (number of fetal deaths ≥28 weeks of gestation per 1000 total births ≥28 weeks of gestation) and neonatal mortality (number of neonatal deaths after live birth before day 28 per 1000 live births). For fetal mortality, we used the lower gestational age threshold of 28 weeks in order to improve comparability between countries. Previous work has shown marked differences in the extent of recording fetal deaths before 28 weeks of gestation in European countries.[20,23]

### Analysis strategy

We first described twinning and higher order pregnancy rates and assessed changes between 2004 and 2010. We then examined geographic patterns of multiple birth rates in 2010 by mapping countries in four groups based on their multiple birth rates. These groups were based on quartiles of the multiple birth rate, rounded to the nearest full percentage in order to have understandable threshold values while maintaining approximately one-quarter of all countries in each group. For descriptive statistics, we present weighted averages and medians for all countries and within these country groups. We used data about maternal age (35 years or more), the use of assisted conception and SET to further explore associations between these variables and multiple birth rates. We calculated Spearman rank correlations to assess the association between these variables and the multiple birth rates in participating countries.

To explore the association between multiple birth rates and outcomes, we calculated risk ratios (RRs) and 95% confidence intervals for multiples compared to singletons for the four perinatal indicators for each country. We used meta-analysis techniques with random effects models to derive pooled RR estimates using the method of DerSimonian and Laird for the entire sample and for the four quartiles of countries. Random-effects measures are interpretable as the association in an average country and relevant for inferences for the population of countries. We also calculated population attributable risks (PARs) and their 95% confidence intervals for multiples to assess the contribution of multiplicity to newborn outcomes at the population level overall and for each of the four groups. Analyses were carried out with Stata v13.0.

## Results

### Multiple births

The median twinning rate in Europe was 16.8 twin births per 1000 women having live or stillbirths in 2010, as shown in Table 1. There was significant variation between countries. The lowest rates were observed in Romania (9.0/1000), Latvia (12.6/1000) and Lithuania (12.9/1000), and the highest in Cyprus (25.1/1000), Brussels (22.6/1000), Czech Republic (21.0/1000) and Denmark (20.9/1000).

**Table 1:** Multiple birth rates (MBR) in participating countries in 2010.

Multiple birth rate group	Country	Total births	Multiples	Multiple rate per 1000	Twinning rate per 1000	Triplet+ rate per 1000
1. Lowest MBR <15 ‰	Romania	213 053	1 910	9.1	9.0	0.2
	Latvia	19 003	241	12.7	12.6	0.1
	Lithuania	30 568	401	13.1	12.9	0.3
	Poland	409 372	5 591	13.7	13.4	0.3
	Iceland	4 834	69	14.3	14.3	0.0
	Sweden	113 488	1 622	14.3	14.0	0.3
	Slovakia	55 012	808	14.7	14.5	0.2
2. MBR 15–16 ‰	Estonia	15 646	234	15.0	14.7	0.3
	Portugal	100 229	1 539	15.4	15.1	0.2
	Finland	60 421	937	15.5	15.3	0.2
	UK combined	799 286	12 546	15.7	15.5	0.2
	Italy	537 633	8 452	15.7	15.0	0.7
	Norway	61 539	1 029	16.7	16.4	0.4
3. MBR 17–18 ‰	Ireland	74 313	1 272	17.1	16.8	0.3
	Austria	77 592	1 366	17.6	17.2	0.4
	France	796 066	14 100	17.7	17.4	0.3
	Netherlands	175 871	3 164	18.0	17.7	0.3
	Luxembourg	6 440	119	18.5	18.3	0.2
	Switzerland	78 784	1 470	18.7	18.4	0.3
	Slovenia	22 000	411	18.7	18.5	0.2
	Germany	625 615	11 819	18.9	18.5	0.4
4. Highest MBR ≥19 ‰	Belgium combined	130 925	2 526	19.3	19.0	0.3
	Malta	3 952	80	20.2	18.7	1.5
	Spain	478 037	9 846	20.6	20.2	0.4
	Denmark	62 203	1 304	21.0	20.9	0.1
	Czech Republic	114 406	2 419	21.1	21.0	0.1
	Cyprus	8 355	222	26.5	25.1	1.4
All countries	Weighted average	5 074 643	85 497	16.8	16.5	0.3
	Median	27 countries		17.1	16.8	0.3

NOTE: Countries are ordered by MBR. Data from Cyprus are from 2007.

The twinning rate increased on average by 1.7 percentage units (+12.5%) between 2004 and 2010. Compared to 2004, the twinning rate decreased only in the Netherlands, Norway and Denmark (-2.1 percentage units, -10.5%) which had relatively high twinning rates in 2004. The rate changed very little in Sweden, Northern Ireland and Finland (+0.3 percentage units, +2.3%). In all other 22 European countries the increase was substantial (+2.4 percentage units, +17.0%). The biggest increases were in Brussels (6.2 percentage units), Malta (5.7 percentage units), and Luxembourg (4.8 percentage units) (S1 Table).

The median triplet rate in Europe in 2010 was 0.3 per 1000 women delivering one or more live or stillbirths. Overall the triplet rate was similar in different parts of Europe. The highest triplet rates were in Brussels (0.6/1000), Italy (0.7/1000), Cyprus (1.4/1000) and Malta (1.5/1000), even though the numbers of triplets were very low in some participating countries. As expected, the triplet rates correlated with twinning rates (r = 0.47, p = 0.006, n = 32).

**Fig 1:**
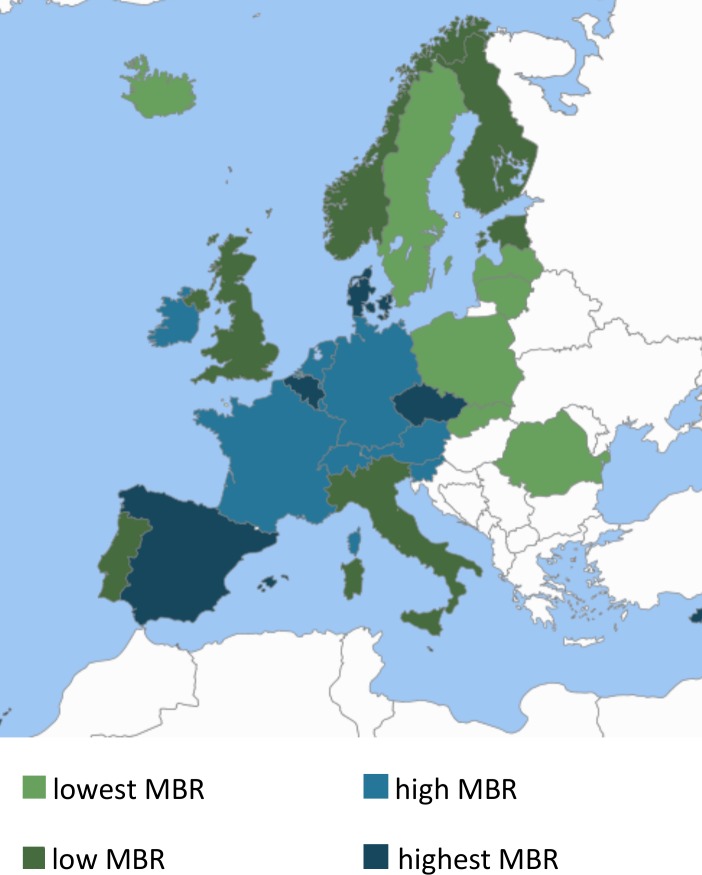
Multiple birth rates (MBR) for selected European countries by MBR group, 2010.

Fig 1 maps the variation in multiple birth rates in 2010 across Europe based on the four groups of countries defined by their multiple birth rates. The countries in the lowest group (less than 15 per 1000) included Eastern and Central European countries (Romania, Latvia, Lithuania, Poland, and Slovakia) as well as some Nordic countries (Iceland and Sweden). The second group (15.0 to 16.9 per 1000) included Estonia, Portugal, Finland, the UK, Italy, Norway, and Ireland, and the third group (17.0 to 18.9 per 1000) Austria, France, Netherlands, Luxembourg, Switzerland, Slovenia, and Germany. Belgium, Malta, Spain, Denmark, Czech Republic, and Cyprus were in the highest group (≥19 per 1000). No clear geographical pattern was observed in the distribution of these groups.

The percentage of women giving birth at ages of 35 or over varied significantly between participating countries, as illustrated in Table 2. It was lowest in Romania (10.9%) and highest in Italy (34.7%). Within the four multiple birth rate groups, the percentage of women 35 years or more when delivering was lowest in the countries with multiple birth rates less than 15 per 1000 deliveries (median 14.7%), but there were no clear differences between the three other groups (medians of 20.2%, 22.4% and 18.2%). Overall, however, the multiple birth rate weakly correlated with the proportion of women giving birth at age 35 and over (rho = 0.34, p = 0.08, n = 28). In contrast, the multiple birth rate was not correlated with the percentage of newborns following assisted conception (r = 0.27, p = 0.28, n = 17) or the proportion of SET (r = -0.14, p = 0.58, n = 17).

**Table 2:** Maternal age and use of assisted conception and SET in participating countries in 2010, with countries ordered by multiple birth rate (MBR) in 2010.

Multiple birth rate group	Country	Maternal age ≥35 (%)	Babies born after assisted conception as a percentage of all births (ESHRE) (%)	SET IVF+ICSI (ESHRE) (%)
1. Lowest MBR <15 ‰	Romania	10.9	-	7.5
	Latvia	14.7	-	-
	Lithuania	14.9	-	6.4
	Poland	11.8	0.8	20.3
	Iceland	19.1	4.4	42.5
	Sweden	22.5	3.5	73.3
	Slovakia	12.6	-	-
2. MBR 15–16 ‰	Estonia	20.7	-	-
	Portugal	21.7	1.9	19.7
	Finland	18.0	3.0	67.5
	UK combined	19.7	2.2	29.9
	Italy	34.7	1.7	19.2
	Norway	19.5	4.1	-
3. MBR 17–18 ‰	Ireland	27.9	1.2	28.0
	Austria	19.7	2.0	26.5
	France	19.2	2.0	28.3
	Netherlands	21.6	2.7	-
	Luxembourg	23.3	-	-
	Switzerland	25.8	2.2	18.4
	Slovenia	15.4	5.1	32.2
	Germany	23.6	2.1	14.3
4. Highest MBR ≥19 ‰	Belgium combined	23.2	4.0	50.4
	Malta	15.5	-	-
	Spain	29.5	2.8	17.4
	Denmark	20.9	5.9	45.2
	Czech Republic	15.4	-	-
	Cyprus	15.5	-	-
All countries	Weighted average	21.8		
	Median	19.7		

NOTE: ESHRE data collection is based on the year of treatment and it only includes countries with complete data available. In ESHRE data babies born after assisted conception include children born as a result of IVF (In Vitro fertilisation), ICSI (Intracytoplasmic sperm injection), FET (Frozen embryo transfer) procedures with own gametes and ED (egg donation). Total rate for babies born after assisted conception as a percentage of all national births (%) is given only for countries where all data were reported. Euro-Peristat data from Cyprus are from 2007.

### Preterm birth

Among the 31 countries/regions providing data on preterm birth in 2010, the median preterm birth (<37 weeks) rate for singletons was 5.6% and for multiples 53.4% (Table 3). Median rates of very preterm birth (<32 weeks) were 0.7% and 8.8% for singletons and multiples, respectively. Average rates were similar to the medians. The proportion of preterm multiple births was the highest in Cyprus (66.9%), Austria (66.0%) and Portugal (63.1%), and lowest in Latvia (39.6%), Denmark (41.6%) and France (42.1%). The proportion of very preterm multiple births was the highest in Austria (12.4%), Slovenia (12.4%), Northern Ireland (11.9%) and Germany (11.0%), and the lowest in Malta (5.6%), Latvia (5.9%) and Cyprus (6.7%).

**Table 3:** Multiple birth rates (MBR) and risk ratios of very preterm birth and overall preterm birth by multiplicity in participating countries in 2010.

		Total		Very preterm birth <32 weeks			Preterm birth <37 weeks		
Multiple birth rate group		Number of live births		Singletons	Multiples	Risk ratio	Singletons	Multiples	Risk ratio
	Country	Singletons	Multiples	%	%	RR (95% CI)	%	%	RR (95% CI)
Lowest MBR<15 ‰	Romania	208 325	3 874	1.1	7.2	6.6 (5.9–7.5)	7.6	42.7	5.6 (5.4–5.9)
	Latvia	18 662	477	1.0	5.9	6.2 (4.2–9.1)	4.9	39.6	8.1 (7.1–9.2)
	Lithuania	30 035	796	0.7	8.2	11.1 (8.5–14.6)	4.3	47.9	11.1 (10.1–12.1)
	Poland	402 171	11 124	0.8	8.1	10.4 (9.7–11.2)	5.3	52.6	10.0 (9.7–10.2)
	Iceland	4 739	133	0.4	9.8	24.4 (12.3–48.3)	4.1	44.4	10.8 (8.5–13.6)
	Sweden	111 474	3 232	0.6	8.5	13.1 (11.5–15.0)	4.7	45.5	9.6 (9.2–10.1)
	Slovakia	54 041	1 604	0.8	9.2	11.9 (10.0–14.3)	5.7	51.8	9.0 (8.5–9.6)
	Pooled RR					10.1(8.1–12.5)			8.9 (7.3–10.9)
MBR 15–16 ‰	Estonia	15 354	459	0.9	7.6	8.8 (6.1–12.6)	4.6	42.7	9.4 (8.2–10.6)
	Portugal	98 207	3 077	0.7	10.1	13.5 (11.8–15.3)	5.9	63.1	10.6 (10.2–11.0)
	UK: Northern Ireland	25 504	771	0.9	11.9	14.5 (11.5–18.3)	5.6	58.2	10.9 (10.1–11.8)
	Finland	59 273	1 873	0.5	8.2	15.3 (12.7–18.5)	4.3	46.8	10.8 (10.2–11.5)
	UK: England and Wales	689 420	21 945	0.9	10.1	10.9 (10.4–11.4)	5.6	53.4	9.5 (9.3–9.6)
	Italy	523 153	17 022	0.7	8.9	12.0 (11.4–12.7)	5.7	58.6	10.2 (10.1–10.4)
	UK: Scotland	55 343	1 784	0.9	10.6	12.2 (10.4–14.3)	5.5	53.6	9.7 (9.2–10.3)
	Norway	60 131	1 981	0.7	8.8	12.7 (10.7–15.0)	4.9	48.7	9.9 (9.4–10.5)
	BE: Wallonia	36 882	1 263	0.7	8.6	13.0 (10.5–16.2)	6.5	61.7	9.5 (9.0–10.1)
	Pooled RR					12.4 (11.4–13.5)			10.1 (9.7–10.6)
MBR 17–18 ‰	Ireland	72 699	2 536	0.7	8.6	12.0 (10.3–14.0)	4.2	48.5	11.5 (10.9–12.1)
	Austria	75 950	2 748	0.9	12.4	13.8 (12.2–15.7)	6.3	66.0	10.4 (10.0–10.8)
	France	14 279	435	0.6	7.1	12.3 (8.2–18.3)	5.5	42.1	7.7 (6.7–8.7)
	Netherlands	170 404	6 033	0.8	9.6	11.5 (10.5–12.6)	5.9	54.3	9.3 (9.0–9.5)
	Luxembourg	6 285	234	0.6	10.3	16.1 (9.9–26.3)	6.3	57.7	9.2 (8.0–10.6)
	Switzerland	76 975	2 915	0.7	8.9	13.2 (11.4–15.3)	5.2	55.2	10.5 (10.1–11.0)
	Slovenia	21 482	816	0.8	12.4	15.8 (12.5–20.1)	5.5	52.9	9.6 (8.9–10.5)
	Germany	611 864	23 697	0.9	11.0	11.6 (11.1–12.1)	6.5	57.9	9.0 (8.8–9.1)
	Pooled RR					12.6 (11.7–13.6)			9.7 (9.0–10.4)
Highest MBR ≥19 ‰	BE: Flanders	67 029	2 608	0.7	10.5	15.3 (13.3–17.7)	6.0	57.2	9.5 (9.1–10.0)
	Malta	3 856	162	0.6	5.6	8.9 (4.2–18.9)	5.4	49.4	9.1 (7.4–11.2)
	Spain	382 136	16 778	0.8	8.3	10.5 (9.9–11.2)	5.9	53.8	9.0 (8.9–9.2)
	Denmark	60 667	2 606	0.7	7.5	10.3 (8.7–12.1)	4.9	41.6	8.6 (8.1–9.1)
	Czech Republic	111 616	4 783	0.7	8.7	12.5 (11.2–14.1)	6.1	53.7	8.8 (8.5–9.1)
	BE: Brussels	23 662	1 129	1.0	9.6	9.4 (7.6–11.7)	6.2	55.0	8.9 (8.3–9.6)
	Cyprus	8 067	450	0.8	6.7	8.0 (5.3–12.2)	7.2	66.9	9.2 (8.3–10.2)
	Pooled RR					11.3 (10.0–13.0)			9.4 (9.1–9.8)
All countries	Weighted average	4 099 532	139 339	0.8	9.4		5.8	54.4	
	Median	31 countries		0.7	8.8	31 countries	5.6	53.4	
	Pooled RR					11.7 (11.0–12.4)			9.4 (9.1–9.8)

NOTE: Data from France are from a nationally representative survey. Data from Cyprus are from 2007.

Multiples had a nine-fold relative risk (pooled RR 9.4, 95% Cl 9.1–9.8) of preterm birth compared with singletons (Table 3). The lowest RR was observed in Romania (5.6, 95% CI 5.4–5.9) and the highest in Ireland (11.5, 95% CI 10.9–12.1). The risk of very preterm births was 12 times higher in multiples (Pooled RR 11.7, 95% CI 11.0–12.4) but a substantial variation between countries was observed, ranging from the lowest in Latvia (RR 6.2, 95% CI 4.2–9.1) to the highest in Iceland (RR 24.4, 95% CI 12.3–48.3). The pooled RRs within the four groups defined by the multiple birth rates did not differ significantly from the overall pooled RR for preterm birth or very preterm birth.

### Fetal and neonatal mortality

The median fetal mortality rate at or after 28 weeks among singletons was 2.8 per 1000 total births among the 30 countries/regions that provided these data (Table 4). The highest rates were in France (4.1/1000), Latvia (4.0/1000), Brussels (3.9/1000) and Romania (3.8/1000). Among multiples, the median fetal mortality rate was 7.0 per 1000 total births with the highest rates in Wallonia (16.0/1000), Romania (15.0/1000), Malta (12.5/1000) and France (11.4/1000). Iceland had no fetal deaths at 28+ weeks among multiples in 2010. The estimated pooled risk ratio for fetal death among multiple births compared with singletons was 2.4 (95% Cl 1.5–3.6). Within countries, the highest risk ratio was observed in Wallonia (RR 5.7, 95% CI 3.6–9.2) and the lowest in Estonia (RR 0.8, 95% CI 0.1–5.8).

**Table 4:** Multiple births rates (MBR) and rate ratios of fetal and neonatal mortality by multiplicity in participating countries in 2010.

		Total		Fetal mortality ≥ 28 weeks			Neonatal mortality		
		Number of births		Singletons	Multiples	Risk ratio	Singletons	Multiples	Risk ratio
Quartile of multiple birth rates	Country	Singletons	Multiples	per 1000 total births	per 1000 total births	RR (95% CI)	per 1000 live births	per 1000 live births	RR (95% CI)
Lowest MBR <15 ‰	Romania	209 120	3933	3.8	15.0	4.0 (3.0–5.2)	4.5	26.8	6.0 (4.9–7.3)
	Latvia	18 764	484	4.0	8.5	2.1 (0.8–5.7)	3.2	14.7	4.6 (2.1–9.9)
	Lithuania	30 167	810	3.3	7.6	2.3 (1.0–5.3)	2.3	15.1	6.4 (3.5–11.9)
	Poland	403 781	11 234	2.9	6.9	2.4 (1.9–3.0)	3.2	15.3	4.8 (4.1–5.7)
	Iceland	4 765	138	1.9	0.0	1.9 (0.1–33.0)	0.6	22.2	35.6 (7.3–174.9)
	Sweden	111 721	3286	2.7	5.8	2.3 (1.4–3.6)	1.3	9.6	7.2 (4.9–10.5)
	Slovakia	54 204	1621	2.9	8.9	3.0 (1.8–5.2)	1.4	16.2	11.5 (7.4–17.9)
	Pooled RR					2.8 (2.2–3.5)			6.7 (5.1–8.8)
MBR 15–16 ‰	Estonia	15 412	472	2.7	2.2	0.8 (0.1–5.8)	1.8	4.4	2.4 (0.6–10.0)
	Portugal	98 690	3100	2.3	5.6	2.5 (1.5–4.0)	1.4	9.4	6.7 (4.5–10.0)
	UK: Northern Ireland	24 902	790	3.3	8.0	2.3 (1.0–5.4)	3.1	25.6	8.7 (5.3–14.2)
	Finland	59 484	1887	1.9	5.4	2.9 (1.5–5.6)	1.2	11.7	9.9 (6.2–16.0)
	UK: England and Wales	699 494	22 431	3.6	8.3	2.3 (2.0–2.6)	2.2	12.4	5.6 (4.9–6.3)
	Italy	529 226	17 293	2.2	6.8	3.0 (2.5–3.7)	1.4	11.4	8.2 (7.0–9.6)
	UK: Scotland	57 860	1813	3.5	7.4	2.2 (1.2–3.8)	2.1	14.6	6.8 (4.5–10.4)
	Norway	60 831	2081	2.1	7.6	3.6 (2.1–6.2)	1.7	10.2	6.2 (3.9–9.9)
	BE: Wallonia	37 133	1297	2.8	16.0	5.7 (3.6–9.2)	1.5	19.0	12.5 (7.8–20.1)
	Pooled RR					2.6 (2.2–3.0)			7.0 (5.7–8.5)
MBR 17–18 ‰	Ireland	73 041	2554	3.7	3.6	1.0 (0.5–1.9)	1.8	10.3	5.6 (3.7–8.5)
	Austria	76 226	2763	2.4	4.5	1.9 (1.0–3.4)	1.7	15.6	9.4 (6.6–13.2)
	France	14 455	443	4.1	11.4	2.8 (1.1–6.9)	NA	NA	-
	Netherlands	172 707	6131	2.6	10.3	3.9 (3.0–5.1)	2.9	14.6	5.0 (4.0–6.2)
	Luxembourg	6 321	239	2.9	4.4	1.5 (0.2–11.5)	0.6	25.6	40.3 (11.4–141.8)
	Switzerland	77 314	2962	1.9	8.8	4.7 (3.1–7.1)	1.9	18.5	9.5 (7.0–12.9)
	Slovenia	21 589	827	3.2	6.3	2.0 (0.8–4.9)	1.3	14.7	11.3 (5.8–22.1)
	Germany	613 796	23 868	2.2	4.4	2.0 (1.7–2.5)	NA	NA	-
	Pooled RR					2.4 (1.7–3.5)			8.4 (5.8–12.3)
Highest MBR ≥19 ‰	BE: Flanders	67 330	2646	2.6	8.6	3.3 (2.1–5.1)	1.8	13.8	7.5 (5.2–10.9)
	Malta	3 872	164	3.1	12.5	4.0 (0.9–17.8)	4.4	6.2	1.4 (0.2–10.5)
	Spain	383 262	16 891	2.5	5.9	2.3 (1.9–2.9)	NA	NA	-
	Denmark	60 899	2614	2.3	2.0	0.8 (0.3–2.1)	1.6	8.4	5.1 (3.2–8.1)
	Czech Republic	112 116	4804	1.4	1.7	1.2 (0.6–2.4)	1.4	8.6	6.3 (4.5–8.9)
	BE: Brussels	23 932	1149	3.9	7.2	1.8 (0.9–3.8)	2.6	5.3	2.0 (0.9–4.7)
	Cyprus	8 120	461	NA	NA	-	NA	NA	-
	Pooled RR					2.4 (1.5–3.6)			5.9 (3.8–9.0)
All countries	Weighted average	4 128 350	141 156	2.9	7.0		2.1	13.9	
	Median	30 countries		2.8	7.0	27 countries	1.8	14.6	
	Pooled RR					2.4 (1.5–3.6)			7.0 (6.1–8.0)

NOTE: Gestational age = best obstetric estimate in completed weeks. Data from France are from a nationally representative survey. Data from Cyprus are from 2007.

Among singletons, the median neonatal mortality rate was 1.8 per 1000 live births in the 27 countries/regions with these data (Table 4). The median neonatal death rate among multiples was 14.6 and was highest in Romania (26.8/1000), Luxembourg (25.6/1000), Northern Ireland (25.6/1000) and Iceland (22.2/1000) and lowest in Estonia (4.4), Brussels (5.3/1000), Malta (6.2/1000) and Denmark (8.4/1000). Compared to singletons, twins had a seven-fold risk of neonatal death (Pooled RR 7.0, 95% Cl 6.1–8.0). Excluding Luxembourg, Iceland and Malta with small numbers, the highest risk was observed in Wallonia, Slovakia and Slovenia (11–12-fold) and the lowest in Estonia and Brussels (2-fold). The pooled RR for fetal and neonatal mortality did not differ between the four groups.

### Population attributable risks (PAR)

**Fig 2:**
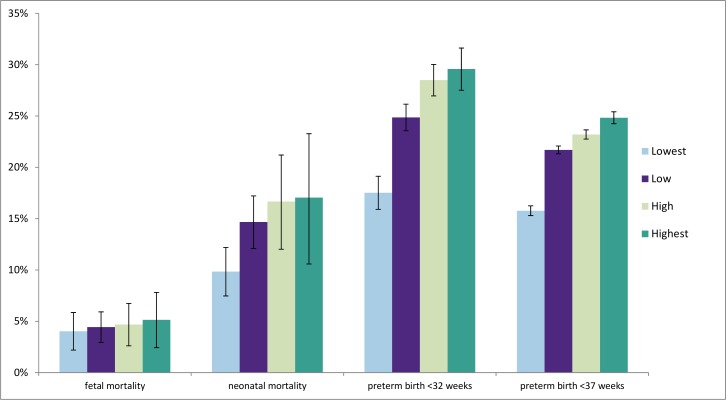
Percentage of four perinatal outcomes attributable to multiple pregnancy in four groups of countries defined by their multiple birth rates in 2010.

The population risks of fetal death, neonatal death, preterm and very preterm birth attributable to multiple births are shown in Fig 2. For preterm birth (<37 weeks), the total PAR was 21.6% (range between four groups 15.8–24.8) and for very preterm births (<32 weeks) it was 25.3% (range between four groups 17.5–29.6). The PARs for preterm birth were higher than for fetal and neonatal mortality: the total PAR for fetal mortality at 28+ weeks was 4.4% (range between four groups 4.0–5.1), and for neonatal deaths 13.4% (range between four groups 9.8–17.1). When comparing countries using the four multiple birth rate groups, the PARs for all the four outcomes (preterm, very preterm, fetal and neonatal mortality) increased from the countries in the lowest to the highest multiple birth rate groups. Significant differences were observed between the four groups for preterm births (<37 weeks between all groups and <32 weeks between all groups except the third and the fourth group). For neonatal mortality only the first group and second group (low) were significantly different from the others.

## Discussion

Our data showed wide variation in multiple birth rates and trends over time in Europe, with no straightforward pattern between geographical areas. Older women are known to be more likely to have spontaneous multiple pregnancies and to use ART [3, 24] but the association of multiple birth rates with maternal age in our data was not very strong. Some countries with similar proportions of older mothers had widely differing rates of multiple births as, for instance, Sweden and Iceland, compared to Spain and Denmark. This variation suggests that other population and policy factors play a role in determining the multiple birth rate and that countries could take further measures to reduce these rates. Our data also showed that multiples continue to face significantly higher risks of adverse perinatal outcomes and illustrated their impact at the population-level.

There were no significant correlations between the multiple birth rates and ESHRE data on the proportion of ART births or SET. The ESHRE data underscore, however, the substantial differences between countries in the proportion of births from ART (with a range from 0.8% in Poland to 5.9% in Denmark) as well as SET (from 6.4% in Lithuania to 73.3% in Sweden)[22] which highlights the differences in clinical practices[25, 26]. Other comparative cross-country research has found an association with the use of ART and SET and the proportion of multiples due to ART, which is a more specific outcome measure.[25] Several reasons could also explain the lack of association in our study. Data were not available for all countries, and those available may not have national coverage; the proportion of ART births and SET could not be taken into account simultaneously to assess the role of ART treatments. In addition we could not take into account the use of ovulation induction, as comparable data on this practice in European countries are not available.[23] Finally we were not able to simultaneously adjust for maternal age.

This research documents the high risks of very preterm birth, preterm birth and neonatal and fetal mortality faced by infants from multiple pregnancies and confirms previous studies.[27–29] Rate ratios for these outcomes for multiples compared to singletons differed across countries, but there were no differences between country groups defined by the multiple birth rates. Previous research by Euro-Peristat on 12 countries using data from 2000 found fairly similar risks associated with multiple pregnancy for fetal mortality (OR 3.0), but a lower overall risk of neonatal mortality (4.9 compared to 7 in this study).[30] However, those data were adjusted for maternal age and parity which may have reduced differences. Furthermore, neonatal mortality has declined more over this period than fetal mortality and this may contribute to changes in relative risks.[18] The Euro-Peristat project also studied the association between multiple pregnancies and preterm birth in 2000 in 11 countries and documented PARs for multiple births between 18% and 25% for preterm birth, which is concordant with the 21.7% observed in this study.[31]

Country comparisons make it possible to assess both differences in practices and outcomes and help us to highlight areas for development and to advance use of best practices across Europe. The wide variation in multiple birth rates as well as their contribution to national rates of preterm delivery and neonatal mortality suggest that many countries could act to bring down their rate of multiple pregnancies and in so doing improve child health outcomes. We found substantial differences in PAR between countries based on their multiple birth rates for preterm birth and neonatal death, illustrating both the short term and long term population impacts that these policies could have by bringing down neonatal rates and reducing risk for longer term adverse neurodevelopmental outcomes associated with preterm birth. This study also illustrates the challenges of finding direct links between ART policies and outcomes–due, in part, to the availability of data on the use of ART and variations in medical practices, but also to the need to take into consideration other contextual factors–such as population factors and health system factors, including financing.[32]

A strength of this study is its broad geographic coverage and use of common protocols to collect data and derive indicators for the participating countries. Differences in data sources and definitions complicated comparisons, however, and these challenges should be taken into account when reviewing the European situation. A majority of countries (19 in 2010) used some linkage procedures to merge data from different sources to increase the availability of data and their quality. Use of linkage could serve to integrate information from ART registers into birth registers and thereby improve data availability and quality. We were also limited because our analyses were based on aggregated data. Having anonymized individual patient data on core perinatal data items for every newborn in Europe would enable the derivation of more detailed statistics and improved possibilities for research, but data protection laws and their interpretations may make this impossible. Finally, the definition of fetal deaths complicated comparisons. In some countries late terminations of pregnancies at or after 22 weeks are recorded as fetal deaths and in these cases they are included in the fetal death rate. Comparing fetal death rates at or after 28 weeks largely overcomes these problems because the number of terminations at this point of pregnancy is very low [20, 33]. On the other hand, this leaves us unable to assess the association between multiple birth rates and early fetal mortality. For neonatal mortality, we did not remove births at 22 to 23 weeks, although Euro-Peristat has done this in previous studies.[20] This would have eliminated countries because fewer countries are able to produce gestational specific neonatal than fetal mortality.

## Conclusion

Many countries could take further measures to reduce their rates of multiple births. Such efforts could improve perinatal outcomes leading to better short and long-term child health at the population level.

## Supporting Information

S1 Table Multiple birth rates in the participating countries. (XLSX)
